# As good as it gets? A new approach to estimating possible prediction performance

**DOI:** 10.1371/journal.pone.0296904

**Published:** 2024-10-16

**Authors:** David Anderson, Margret Bjarnadottir

**Affiliations:** 1 Villanova School of Business, Villanova, PA, United States of America; 2 Robert H. Smith School of Business, University of Maryland, College Park, MD, United States of America; Univerzitet Singidunum, SERBIA

## Abstract

How much information does a dataset contain about an outcome of interest? To answer this question, estimates are generated for a given dataset, representing the minimum possible absolute prediction error for an outcome variable that any model could achieve. The estimate is produced using a constrained omniscient model that mandates only that identical observations receive identical predictions, and that observations which are very similar to each other receive predictions that are alike. It is demonstrated that the resulting prediction accuracy bounds function effectively on both simulated data and real-world datasets. This method generates bounds on predictive performance typically within 10% of the performance of the true model, and performs well across a range of simulated and real datasets. Three applications of the methodology are discussed: measuring data quality, model evaluation, and quantifying the amount of irreducible error in a prediction problem.

## 1 Introduction and literature review

How much information does a dataset contain about an outcome of interest? How accurately can the outcome be predicted? This paper develops a method to generate a lower bound on the achievable mean absolute prediction error for a particular outcome and dataset. This minimum achievable error is a measurement of the amount and quality of information contained in the dataset, in relation to the specific outcome. A lower bound on prediction error (or conversely an upper bound on prediction performance) is useful to researchers and practitioners in a number of ways. It can be used as a measure of the quality of information in the dataset, as an objective standard against which to compare the performance of a predictive model, and as an estimate of the predictability of the outcome studied. As such, this paper speaks to the literatures on the quality of information in a dataset and the overall predictability of an outcome. All prediction algorithms are functions that map points in some input space to points on the real number line. If any prediction algorithm is given two observations that are identical in the input space, it must generate identical predictions for those observations. This method builds on the assumption that that if two observations that are very close in the input space are fed into the prediction algorithm, then any prediction algorithm must generate relatively similar predictions for those two observations. The property that small changes in inputs cannot lead to large changes in the predicted outcome, or *internal consistency*, is a key principle underlying this paper. While the structure of some prediction methods, like regression trees, may allow for similar observations to have very different predictions (if, for example, they are just on either side of a split in the tree), typically, no model will be able to consistent detect big changes in the outcome between very similar inputs. Sections 3 and 4 discuss cases where this assumption does not hold. This paper rests on the assumption that an omniscient model, bounded only by the constraints that predictions for similar observations needs to be similar, performs as well, or better than, any internally consistent machine learning model trained on a dataset could do. That is, the resulting error using the omniscient model is a lower bound on the prediction error for any trained machine learning model. Knowing the best possible performance on a dataset allows researchers to quantitatively measure the quality of their datasets before spending time doing model building and evaluation. If the performance of a prediction model is very close to the estimated lower bound, then it is unlikely the model can be improved, whereas if there is a large gap, model refinement may be beneficial. Below, Section 1 reviews the literature on data quality and predictability and highlight how this paper is distinct from and contributes to these two streams of literature. Following, Section 2 describes the methodology in detail, and Sections 3 and 4 show results on simulated and real datasets. The paper concludes with discussion, limitations and conclusions in Sections 5 and 6. The S1 Appendix gives results on additional datasets.

### 1.1 Quality of data

The question of how much relevant information a dataset contains is fundamental to applied statistics as a field. Fisher [1] describes the three problems that statisticians face as problems of specification, estimation, and distribution. However, Mallows [2] describes the “zeroth problem” of applied statistics, which must be addressed before any analysis can be done, as “considering the relevance of the observed data, and other data that might be observed, to the substantive problem. (p. 2)” Without the proper data, no modeling approach will yield acceptable results. However, there is no commonly agreed upon method for defining appropriate and relevant data. Pipino et al. [3] give sixteen dimensions on which data can be judged, including attributes like accessibility, accuracy, and amount of data, all scored on a subjective 0–1 scale. In contrast, the concept of “fitness for use” is a becoming a standard for judging the quality of a dataset [4]. “Fitness for use” is the ability of the data to answer the questions in the specific case that the user requires. Thus, the quality of a dataset is relative to the specific use for which it is intended. Shmueli and Kenett [5] formalize the ideas of data quality and fitness for use into a concept they call Information Quality (InfoQ). Information quality seeks to answer Mallows’ “zeroth problem:” determining how much information a dataset has about a specific outcome of interest. “InfoQ is determined by the quality of its components ‘quality of goal definition’, ‘data quality’, ‘analysis quality’, and ‘quality of utility measure’ and by the relationships between them.” The bounds generated in this paper are a quantitative measure of the “data quality” portion of InfoQ.

### 1.2 Predictability

This methodology further contributes to the research on the predictability of outcomes. Across the social and physical sciences, researchers are interested in what outcomes are relatively more or less “predicatble”. Wright and Goodwin [6] suggest that a result is predictable if well-calibrated estimates for outcome probabilities are able to be generated, and the true underlying probability distribution of the outcome is not too broad. Ehrenberg and Bound [7] claim that in order for a result to meet these conditions, it must have “recurred consistently under a known range of different conditions. (p. 167)” Taleb [8] argues that some outcomes, especially those in the distant future, are essentially unpredictable and unable to be accurately forecasted. Chaotic systems, those with extreme sensitivity to initial conditions, topological mixing, and periodic orbits, are nearly impossible to predict in the long-term [9]. This phenomenon makes it virtually impossible to predict weather more than two weeks into the future. On the other hand, processes that abide by relatively straightforward rules and have direct causes and effects are more likely to be predictable.

There has been research into which outcomes are predictable and which are not in a variety of fields, including health forecasting [10], information systems [11], information security [12], macroeconomics [13], risk management [14], water management [15], and energy demand planning [16–18], among others. These papers discuss individual outcomes or fields, but do not generate a systemic framework for assessing the predictability of a new outcome, or the use of a specific dataset in that prediction.

A stream of prior research has focused on generating bounds on classification performance for specific outcomes or specific algorithms. Seeger [19] derives bounds on minimum classification error for a family of Bayesian Gaussian Process classifiers, under specific assumptions about the data generating process. Rigollet [20] derives error bounds on semi-supervised classification problems, but assumes homogenous clusters in the data. Cover and Hart [21] show that a nearest-neighbor classification model will have less than double the probability of error compared to any other feasible classification model, parametric or not. These previous papers all have focused datasets with specific features, or on specific algorithm performance. In contrast, the bounding method described in this paper is generally applicable both in terms of data types and predictive algorithm used.

In addition to research on bounds of binary classification accuracy, there is also a stream of research using information theory and the idea of entropy to estimate maximum prediction performance on time series data. These papers generate a measure of the entropy in the outcome of some time series (e.g. location, demand, etc.), to estimate how much randomness is in the process. The rest of the time series is then theoretically able to be predicted. Song et. al [22] measured the entropy of human mobility using cell phones, and estimated that only 7% of location changes were completely random or unpredictable. Using this method, Zhao et al. [23] find that up to 83% of New York City taxi demand can be predicted using time series data. Lu et al. [24] show prediction performance of 87% on human mobility predictions using cell phone location data, compared to a theoretical maximum of 88%. In general, these papers find that the entropy-maximum prediction is approachable by machine learning prediction algorithms.

Another stream of work focuses on the prediction errors of individual observations. Bousquet and Elisseeff [25] develop upper bounds on leave-one-out prediction and classification error for any given algorithm, based on its overall performance on a dataset. They base their proofs on the idea of stability; small changes in the inputs of a dataset should not lead to large changes in the predictions of that algorithm. Other authors (e.g., [26–29]), use algorithm stability as a method to generate upper bounds on prediction accuracy for a given algorithm, clustering technique, or as a criteria for model selection. This stability assumption, that small changes in input correspond to at most a small change in output, is the same assumption that undergirds the upper bounding model presented in this paper. Table 1 shows the contribution of this paper in relation to the other most related papers.

**Table 1:** Summary of related approaches.

Paper	Model Type	Input Restrictions	Continuous / Discrete	Bound Error
Seeger [19]	Bayesian Gaussian Process Classifiers	None	Discrete	Up to 25%
Rigollet [20]	Semi-Supervised Classification	Assumes Homogenous Clusters in Data	Discrete	NA
Cover [20]	Nearest Neighbor	Any	Discrete	Asymptotically goes to 50%
Bousquet [25]	SVM	Requiring “Stability” on the inputs	Both	NA
Evgeniou [28]	SVM or Ensembles	Any	Discrete	Up to 40%
This Paper	Any	None	Both	Typically under 20%

This paper offers a new approach to measuring the quality of information in a dataset. Discussions of the bias/variance tradeoff assume there is some reducible error that can be minimized through model complexity and metaparameter tuning [30], and some “irreducible error” which is inherent to the problem and data, and can never be eliminated [31–33]. This contribution of this paper is to give a model-agnostic method to quantify the amount of irreducible error [33] for a given prediction problem. While the idea of irreducible error, or best-possible performance has been widely accepted in the machine learning community, few efforts have been made to directly quantify the amount of this error in a general manner. Our paper is a first attempt to directly quantify this for any data set and for any modelling approach. While the model performs well in a range of situations. In practice, this paper presents a method of generating tighter bounds on a wider range on datasets than the other papers in the literature, at the cost of being a purely data-driven, heuristic approach that offers no performance guarantees or proven theoretical bounds. The limitations section discusses some cases where the method may struggle, due to this lack of theoretical assurance of performance.

## 2 Methodology

The methodology introduced below produces prediction bounds for continuous outcomes, while making no assumptions about the distribution of the underlying process or outcome. As previously stated, the underlying assumption of this paper’s methodology is internal consistency: that if two points are very close to each other in the input space, then they must be mapped to values close to each other in the output space. The choice of distance function is discussed in Section 2.1. The more similar two observations are in the input space, the more similar their predictions must be, and if two observations are identical then they must have identical predictions. This assumption is a reasonable assumption for almost all phenomena studied by the data mining community, and is explicitly true for any parametric or regression-based model. As a numerical example, suppose a researcher is interested in predicting future earnings of college students. In a dataset, there are two students with identical race, gender, major, grade point average (GPA), and SAT score. If this is all of the data she has, she must predict identical salaries for these two students. However, if the first student has an SAT score that is 10 points higher than the second, then it might be reasonable to expect that he will earn a little higher salary, but not dramatically higher. Thus, this method will allow the two predictions to be, for example, $57,000 and $56,000, but not $150,000 and $50,000. If two observations are very similar in the input space, but have very different outcomes, either randomness or an unobserved variable(s) must be causing the differences between the two outcomes. If the two identical students end up taking jobs that pay $90,000 and $50,000, then there is either something random about the salary process, or there are unobserved factors causing this difference. Section 2.1 discusses how to use the data to derive how predictions are allowed to vary based on small changes in the input space. This method adopts the above assumptions and interprets them as constraints on the predictions assigned to similar observations, and finally finds the set of predictions that minimizes the total prediction error over the entire dataset. It generates a “limited omniscient” model that can see all of the true outcomes, but is constrained to generate similar predictions for similar observations. The amount of error generated by the predictions of the omniscient model is the smallest amount of error that any internally consistent predictive model could consistently achieve. Thus, the resulting mean absolute error (MAE) of this model is a lower bound on the achievable MAE of any model. No predictive model that is internally consistent will be able to consistently achieve a lower MAE (better predictive accuracy). While this paper focuses on MAE, the discussion section discusses how it is possible to implement formulations using mean squared error, or any other loss function as an objective.

### 2.1 L.P. formulation

Mathematically, the performance bound can be expressed as the following linear program (LP):

$$\begin{array}{cc} & \underset{P_{i}}{\text{min}}\;\epsilon_{i}^{+}+\epsilon_{i}^{-}\\ \text{Subject}\;\text{to} & \\ & |P_{i}-P_{j}|\leq f\left(D\left(i,j\right)\right)\;\forall \;D\left(i,j\right)\leq \delta \; \end{array}$$ (1)

$$\begin{array}{cc} & P_{i}-Y_{i}+\epsilon_{i}^{+}-\epsilon_{i}^{-}=0\;\forall \;i\; \end{array}$$ (2)

with the following notation:

- *P*_*i*_: The prediction for observation *i*.
- *Y*_*i*_: The true outcome for observation *i*.
-
$\epsilon_{i}^{+}$ and $\epsilon_{i}^{-}$: positive and negative difference between the prediction and the true outcome of observation *i*.
- *D*(*i*, *j*): The distance in the input space between observations *i* and *j*.
- *δ*: A distance threshold, closer than which two observations are considered to be similar.
- *f*(*D*(*i*, *j*)): A function linking distance in the input space and the maximum allowable deviation in predictions.

Constraint (2) ensures that the absolute error $\left(\epsilon_{i}^{+}+\epsilon_{i}^{-}\right)$ for each observation gets added to the objective function. The optimal solution to the LP is the set of predictions that will minimize the sum of these errors. Constraint (1) ensures that if two observations are closer than some threshold, *δ*, then they will have similar predictions. The amount by which two observations are allowed to differ while still considered similar is determined by how far apart they are in the input space, as measured by the distance function *D*. Common distance functions, like the L1 or L2 norm, typically work well in numerical experiments. This paper reports results using the L2 norm. Once a distance function is defined, observations that are closer than the threshold, *δ*, are allowed to differ by no more than some function of that distance, denoted by the linking function *f*.

If *δ* is zero, the LP only has constraints restricting predictions of identical observations. At the opposite extreme, if *δ* is very large, greater than the maximum distance between any two observations, there will be a constraint on the maximum difference in predictions between every pair of observations. Somewhere between these two extremes is a value of *δ* that generates constraints for similar observations, but allows distant observations to vary freely from each other. In Section 3, simulated data are used to generate rules of thumb of how to choose a good value of *δ*. In Section 4,those rules are used to generate bounds on performance for real world datasets.

Without loss of generality, let us assume that the input features are normalized to have a standard deviation of 1. The linking function, *f*(*D*(*i*, *j*)), should be informed by the input data. For example, observations that have a distance of *k* between them (using the distance function chosen above) might be allowed to differ by *β*_max_ × *k*, where *β*_max_ is the largest absolute coefficient in a standardized linear regression model. If a one-standard deviation increase in any input variable is expected to change the outcome variable by no more than *β*_max_, then a total one-standard deviation of distance between two points across all the inputs cannot predictably result in predictions farther apart than *β*_max_. In this paper, *β*_max_ × *D*(*i*, *j*) is used as the linking function, and is demonstrated to generally work well in practice. Section 5 discusses edge cases where it may not work well, and proposes alternative linking functions in those cases.

### 2.2 Measures of predictability and performance

Based on the bounds methodology introduced in the previous section, a statistic, “predictive score” for evaluating a predictive model is defined. Adapting the basic idea of *r*^2^ to the predictive case, and using the estimated upper bound, the predictive score represents the percent of the overall predictable variation that is correctly predicted by a model. The formula is given by:

$$\text{Predictive}\;\text{Score}=1-\frac{{\text{MAE}}_{\text{Model}}-{\text{MAE}}_{\text{Bound}}}{{\text{MAE}}_{\text{Null}}-{\text{MAE}}_{\text{Bound}}}$$

where MAE_Null_ is the mean absolute error from the null model, i.e. predicting the median for each observation, MAE_Model_ is the MAE of the predictive model being evaluated, and MAE_Bound_ is the lower bound on MAE generated by solving the LP formulation above. The null model is simply predicting the median for each observation, as this minimizes the MAE. Section 5 discusses minimizing mean squared error rather than MAE. In that case, the null model would be to predict the mean. The numerator measures the difference between the error of the model and the error of the bound and the denominator measures the total amount of predictable variation. This measure compares how well a predictive model performs relative to the difficulty of a problem or amount of information in the dataset, and is comparable across problems and domains. It can be interpreted as how much room for improvement in the model is theoretically possible, given the data available.

Let us also define a measure, *ϕ*, that quantifies how much of the variability of an outcome variable is possibly predictable by a particular dataset, as:

$$\phi =1-\frac{{\text{MAE}}_{\text{Bound}}}{{\text{MAE}}_{\text{Null}}}$$

Here, the numerator measures how much of the outcome is unpredictable, while the denominator measures the total amount of distance from the median in the outcome. This measure, *ϕ*, is a measure of how much irreducible error is in a prediction problem. A higher *ϕ* value implies that a higher fraction of the total variability in an outcome is irreducible. The ratio of these is the amount of the variation in the outcome that is not possible to predict due to the incompleteness of the data or the inherent randomness of the outcome. Subtracting this ratio from 1 gives us the percentage of the overall variability that is possible to predict; (1 − *ϕ*) of the outcome is unpredictable. If *ϕ* is 1, that means that MAE_Bound_ is 0, meaning that perfect predictive performance may be possible. If *ϕ* is 0 (and MAE_Bound_ = MAE_Null_) it implies the outcome is completely random, or the data available have no information about the outcome.

The next sections provide examples of the methodology applied to simulated and real world datasets, and show how the Predictive Score and *ϕ* can be used to characterize datasets and prediction problems.

## 3 Simulated data

### 3.1 Linear data

This section showcases the performance of the methodology on a range of simulated datasets, emphasizing the accuracy of the performance bounds generated. The benefit of simulated data is the knowledge of the data generating process and therefore the bound can be compared to the true underlying model. The simulations vary the number of observations (*N*), number of explanatory variables (*M*), and amount of noise in the data. For each set of parameters, the mean absolute error of the true model and the minimal mean absolute error suggested by the bounds are measured, which are generated for a range of similarity thresholds, *δ*.

In the example below, each dataset consists of *N* observations of *M* i.i.d. Normal (0, 1) variables, and a Normal (0, *σ*^2^) noise term. Datasets were generated with 100 to 10,000 observations of 3 to 12 covariates, with *σ* ranging from 1 to 4. The outcome variable in each case is the sum of the input variables plus the noise variable. (i.e. *Y* = *X*_1_ + ⋯ + *X*_*M*_ + *σ*). The simulations use *δ* ranging from 0 to 3.5, with the *L*_2_ norm as the distance function. The linking function is *f*(*D*(*i*, *j*)) = 1 × *D*(*i*, *j*). This distance function is appropriate here as a one unit change in any of the input variables leads to a one unit change in the outcome variable. As a result, if the total distance between two observations is *k* units, then their predictions are allowed to vary by no more than *k*.

To evaluate the tightness of each bound, the difference between the fraction of overall variation explained by the true model, and *ϕ*, the fraction predictable indicated by the bound is calculated. Because the data generation process for the simulated data is known, the performance of the true model can be measured, and can be compared to the estimated bound by computing the difference. As an example, if the true model, as defined in the data generating process, predicts 73% of the total deviation in the outcome, whereas the generated bound indicates that up to 80% of the deviation is predictable, the looseness would be 7%, or 0.07. The closer the looseness is to zero, the more accurate the bound. Ideally, for the appropriate selection of *δ*, *D* and *f*, this difference would be zero. A negative looseness would imply that the generated bound on the error is smaller than the error of the true model.

**Fig 1:**
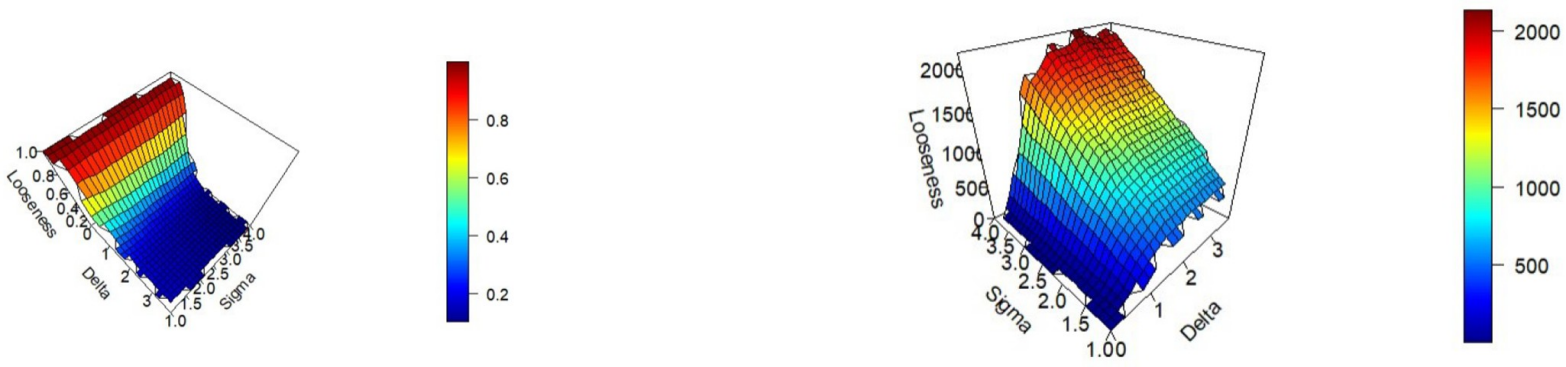
The performance bound (a) and the Looseness (b) as a function of *δ* and *σ* when N = 750 and M = 4.

**Fig 2:**
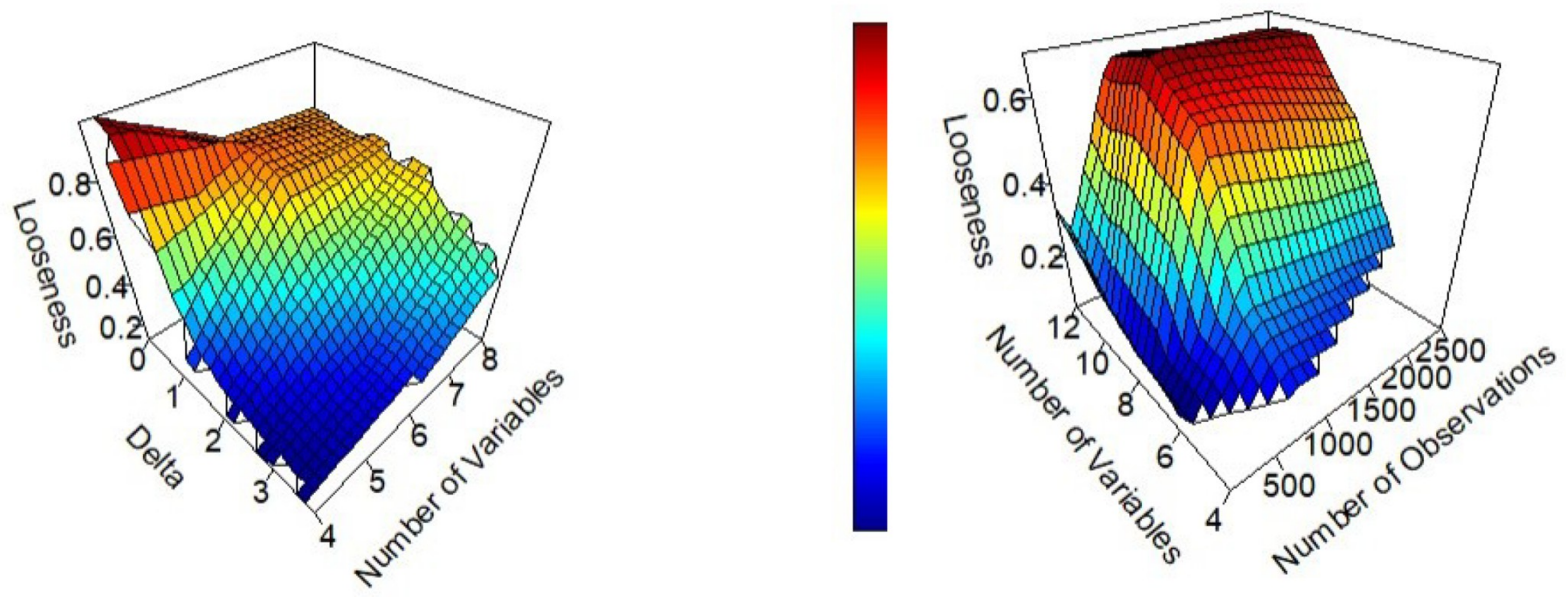
Looseness as a function of (a) *δ* and M (N = 750, and *σ* = 3) and (b) N and M (*σ* = 3, and *δ* = 3).

The results of the simulations are summarized in Figs 1 and 2 below. Fig 1(a) shows the performance bound when *N* equals 750, and *M* equals 4, as the “closeness” threshold (*δ*) and amount of noise in the dataset (*σ*) are varied. In general as noise is increased in a prediction scenario the predictive performance deteriorates. This fact is reflected in the bound. For a given value of *δ*, the bound on MAE increases as a function of *σ*, that is, as the level of noise increases so does the bound on achievable MAE. Further, for a given level of *σ*, as *δ* increases so does the bound on achievable MAE, that is as the predictions of more and more pairs of observations are restricted to be similar, the bound increases. There are some boundary conditions. When *σ* is small, the observable input features do a good job of explaining the variation in the outcome, and bound on the error is very low, although increasing in *δ*. Similarly, if *δ* is small, very few constraints are added to the model, and thus the computed performance bound is again low, and remains very low for even high values of *σ*. When both *σ* and *δ* are high, the bound on the prediction performance, MAE, is high. If there is substantial noise in the dataset (*σ* is large), and *δ* is large enough, there are enough constraints in the LP model to enforce similar observations to have similar predictions, reflected by the high bound on the prediction error.

Fig 1(b) shows how the bound performs relative to the true model, as a function of *δ* and *σ*. As expected when *δ* = 0, and only identical observations are restricted to have identical predictions, the looseness of the bound is 1. When *δ* is large enough, (typically above 3 standard deviations in this case), the generated bound is close to the performance of the true model. In this case (*M* = 4, *N* = 750), the average looseness of the bound is approximately 10% or less when *δ* is equal to 2, and is under 7% when *δ* is equal to 3.

The bound on prediction error gets tighter (closer to the performance of the true model) as more constraints are added to the LP, which happens when *N* and *δ* increase. When *δ* increases, more constraints are added because more observations are considered similar. Increasing *N* makes the feature space more densely packed, which means more data points will have close neighbors, and thus more constraints will be added to the LP. The lower bound on prediction error gets looser as more explanatory variables are added. This happens because as the dimension of the input space grows, fewer observations are close to each other, and thus there are fewer constraints in the model; increasing *N* can help counteract this effect. Fig 2 demonstrates this behavior.

The behavior of the bound is studied in 5140 simulation runs, varying *N*, *M*, *σ*, and *δ* as previously described. On average, the bound on MAE ranges from 0% to 40% below the true model’s MAE, with it typically staying within 5% when both *N* and *δ* are sufficiently large.

**Fig 3:**
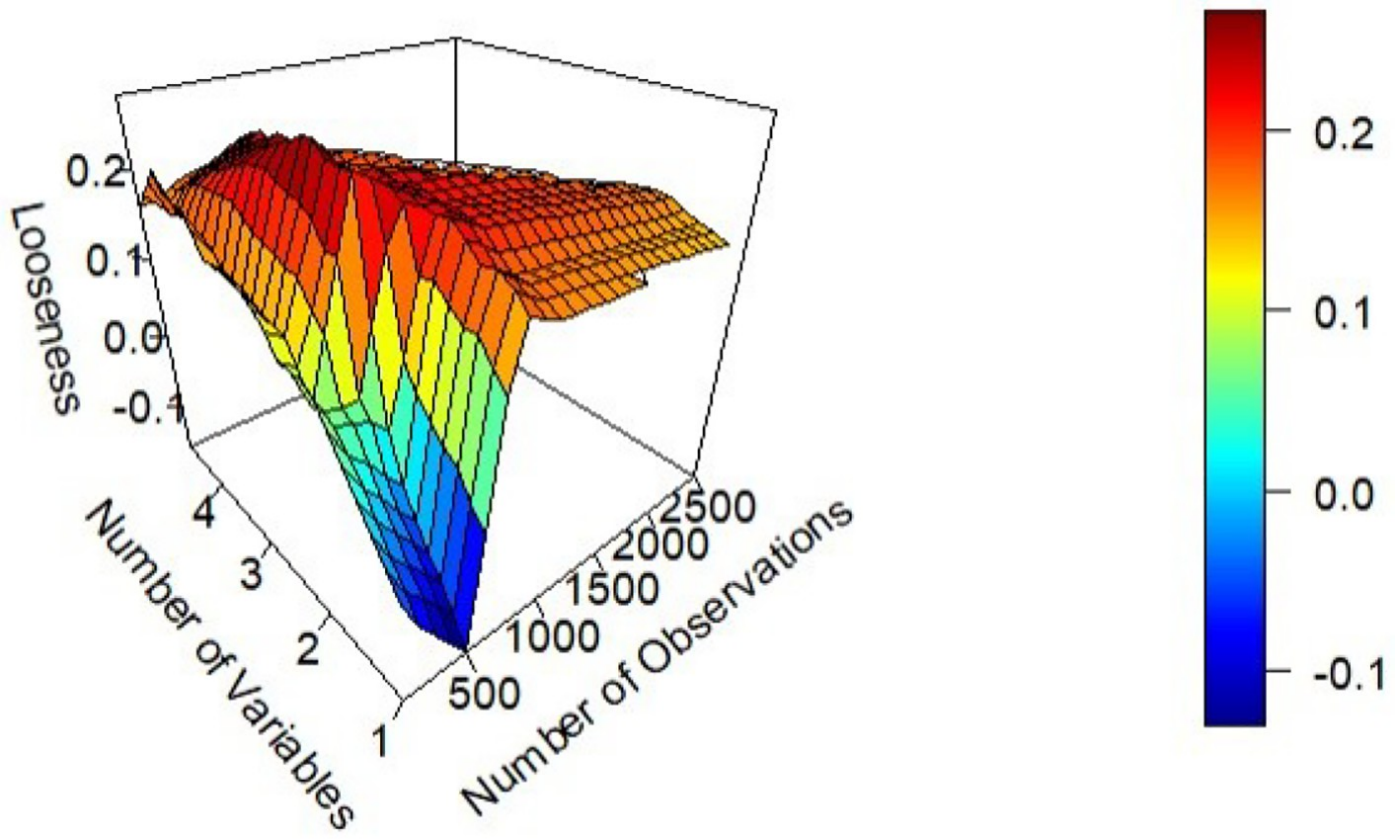
The looseness of the bound compared to the true model, as N and *σ* change, using the 5% rule of thumb to choose *δ*.

The choice of *δ* plays a critical role in determining an appropriate bound. An overly large *δ* becomes restrictive, while an excessively small *δ* produces loose bounds. Based on the simulations, the following rule of thumb emerges: selecting the smallest *δ* that yields 5% of the total possible constraints (and at least an average of 5 constraints per data point, especially in smaller datasets) generally results in a tight bound. When *δ* is arbitrarily large, there will be N * (N-1)/2 constraints in the model, i.e. every pair of points is considered to be close to each other. Following this rule, the average MAE of the bound typically falls within 10% of the true model’s performance. This rule often results in bounds closer to the true model when there is lower noise in the dataset and when *N* is smaller. Nevertheless, it performs consistently well across a wide range of parameter configurations. The least effective application of this rule observed still maintained a looseness below 20%. Fig 3 shows the looseness of the bound as a function of N and *σ* when choosing the smallest *δ* that meets the 5 constraints or 5% rule.

### 3.2 Nonlinear data

**Fig 4:**
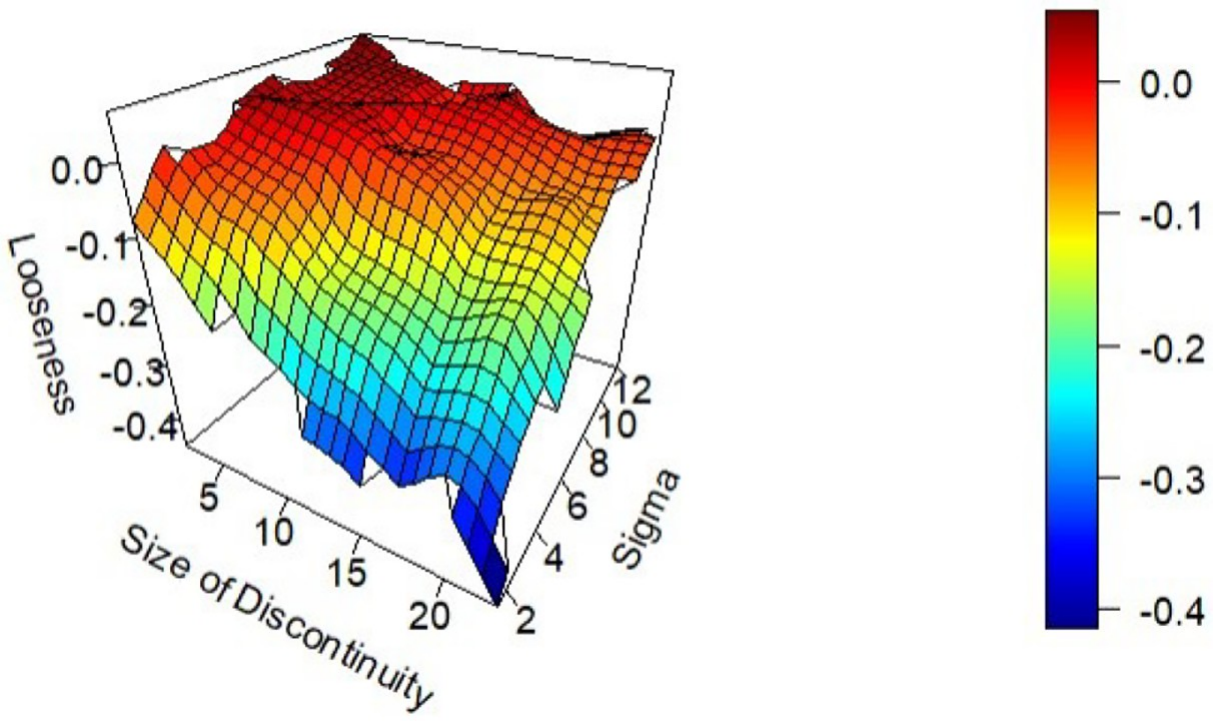
Looseness of the bound as a function of the size of discontinuities in the data and noise.

Section 3.1 demonstrates that the bound works well on a wide range of datasets generated via linear data generating processes. In this section, more complicated datasets are simulated, including quadradic terms, interaction effects, and discontinuities. The bound still performs well, generating tight bounds that are close to the true model’s performance. Again, using the 5% rule for choosing *δ* and using the maximum absolute standardized regression coefficient for the linking function yields bounds that are tight and accurate, when certain broad conditions are met. To investigate the effect of discontinuities in the outcome on the performance of the bound, datasets with discrete jumps in the outcome variable are generated. In this case, four independent predictor variables are generated, and an outcome that is equal to the sum of the four inputs, plus Normally distributed noise, the same as the previous section. However, now a fixed value is added to the outcome for each input variable that is more than 2 standard deviations above the mean. The amount of noise in the dataset and the size of this discontinuity are varied, and bounds on the resulting datasets are generated. Fig 4 shows that the bound performs best, giving the tightest bounds when the discontinuities are small and when there is at least a moderate amount of noise in the data. When there are very large discontinuities in the data, the assumption of internal consistency that the bounding methodology relies upon is violated, and the bound returns a negative looseness. The true model can correctly predict these large discrete jumps, but the locally smooth predictions generated by the bounding algorithm cannot, leading to better predictions than the model claims are theoretically possible.

Next, continuous nonlinear datasets are generated. Again, the datasets have consider four independent input features, but in this scenario, the outcome variable is constructed as the sum of the inputs added to the sum of *α* times the square of the inputs and further added to the sum of *β* times *X*_*i*_ multiplied by *X*_*i*−1_, with an additional Normally generated noise term included.

**Fig 5:**
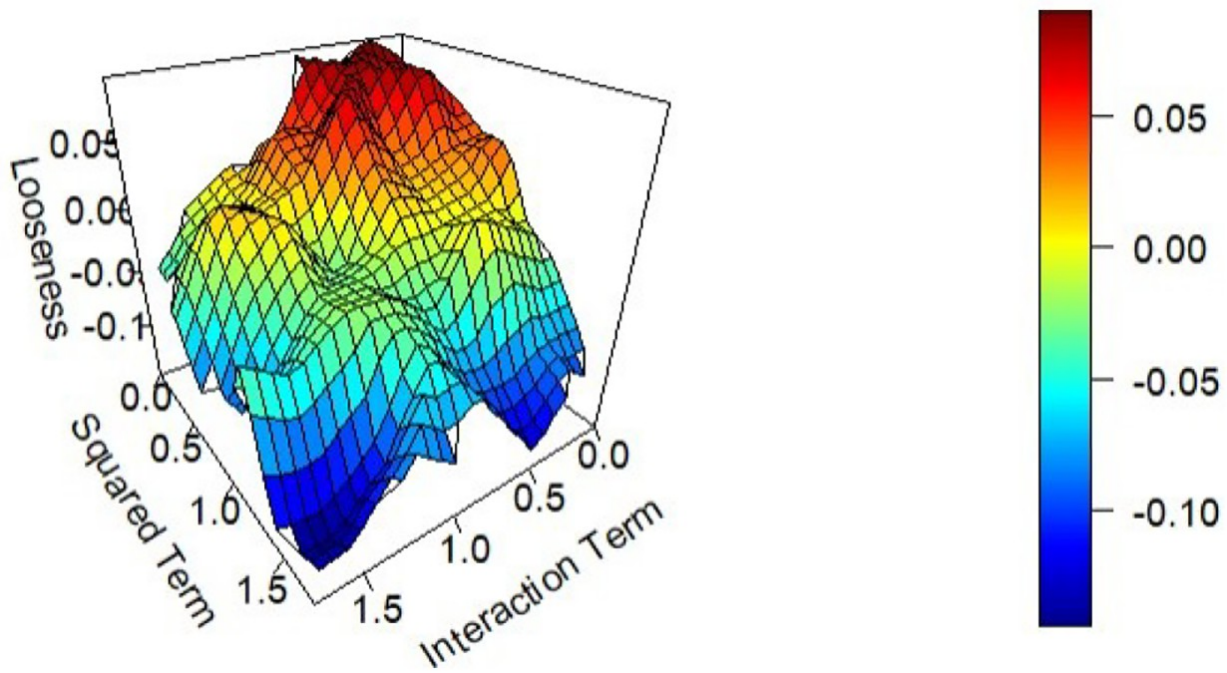
Looseness of the bound as a function of the size of interaction and squared terms in the data generating process.

The weights assigned to the interaction terms and on the squared terms are varied to assess the performance of the bound. Fig 5 presents the results of this analysis.

Moreover, the investigations revealed that when *β*_max_ is considerably large, the resulting constraints appear to be rather lenient. When the model allows a minor alteration in inputs to correspond with a substantial fluctuation in the anticipated outcomes, the solution space for the Linear Program becomes expansive. This extensive solution space often leads to the discovery of solutions with a minimal MAE, which consequently results in more relaxed bounds.

Conversely, in scenarios where *β*_max_ is either moderate or small, and the data doesn’t exhibit significant discontinuities, the formulated bounds exhibit commendable precision. Specifically, these bounds are tight, typically deviating by no more than 10% and notably, they do not tend to exceed their limits.

## 4 Real world data

The following sections generate performance bounds using three real-world datasets. Using the rule of thumb described in Section 3 generates relatively tight bounds compared to the performance of machine-learning models trained on the data. In addition, prediction problems that are intuitively “easier” have lower error bounds compared to problems where the outcome is thought to be less predictable. That is, in problems with have high quality data that contain significant information about the outcome, *ϕ* is high (i.e. the lower bound on MAE is low). In cases where the data is poor, or when the outcome has significant random components, *ϕ* is low (i.e. the lower bound on MAE is high). In this sense, *ϕ* is a measure of both the quality of the dataset, and a way to quantify the relative difficulty of predicting a given outcome.

### 4.1 Technology adoption

In their paper, Agarwal and Karahanna (2000) build and test a theoretical model explaining intention to use the internet as a function of cognitive absorption. Specifically, they hypothesized that perceived ease of use and perceived usefulness are the two main explainers of behavioral intention of internet use. They collected survey data on 288 undergraduate students to measure these constructs and test their relationship. In all, they find significant relationships, with perceived ease of use and perceived usefulness explaining 48% of the variation in behavioral intent.

Using the data from this study, upper bounds on the ability of perceived ease of use and perceived usefulness to predict behavioral intent are generated. The two independent variables and the dependent variable are standardized to a mean of zero and a standard deviation of one. The distance between points is calculated using the L2 norm (using the L1 norm results in almost identical results) and used *f*(*D*(*i*, *j*)) = 0.478 × *D*(*i*, *j*) as the linkage function. This function is chosen because a linear regression of behavioral intent on perceived usefulness and perceived ease of use has the equation: Intent = 0 + 0.306 × Perceived Ease Of Use + 0.478 × Perceived Usefulness. A small change, *ε*, in input features could, therefore, be expected to lead to no more than a 0.478 × *ε* in the outcome.

The null model, predicting the median for each observation, has a MAE of 0.602, compared to 0.528 for the linear regression model reported in the paper. Depending on choice of *δ*, a minimum achievable MAE of 0.138 with *δ* = 0 to 0.388 with *δ* = 0.25 is found. That is, requiring only identical observations to have identical predictions, the minimum achievable MAE is 0.138. Increasing the closeness parameter from 0 to 0.25 (meaning that observations less than 0.25 standard deviations away from each other are considered similar), increases the minimum achievable MAE to 0.388. A value of 0.22 for *δ* is used because it is the smallest value for which the “larger of 5% or 5 constraints per observation” rule of thumb is met.

**Fig 6:**
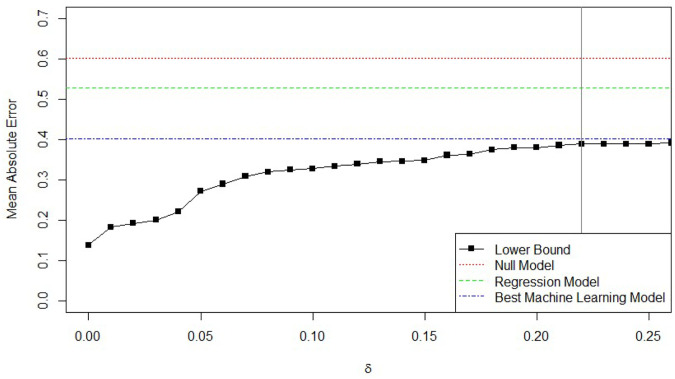
Minimum MAE as a function of *δ* for the technology adoption study.

Using these numbers, $\phi =1-\frac{0.388}{0.602}=0.355$, or that at most 35.5% of the total absolute deviation in the outcome is explainable using the data collected. The other 64.5% of the outcome is either due to inherent randomness, or to unmeasured factors of each subject. Of the 35.5% of the outcome that is predictable, the model reported is able to explain $1-\frac{\left(0.528-0.388\right)}{\left(0.602-0.388\right)}=34.5\%$ of the total variability that is explainable. As there is a large gap between the regression model performance and the theorized optimal performance generated by the upper bound, multiple machine learning algorithms to the data were fit. Both a neural network as well as a single regression tree have better performance than the simple linear regression. The best performance was achieved by a neural network with two hidden layers, of 6 and 3 nodes, respectively, with a MAE of 0.402, corresponding to 93.5% of the total explainable variability. Fig 6 summarizes the performance bound as a function of *δ*, with a vertical line at 0.22, the chosen *δ* value. As *δ* increases further past 0.25, the bound flattens out, because the allowable difference between observations increases as the distance between points grows larger. After a point, the additional constraints added to the LP are so loose that they do not change the optimal solution at all. The neural network model has a 24% lower MAE than the regression model. However, further refining of the model and tuning of the parameters should lead to at most marginal improvements in the performance of this model, as its MAE is now very close to the generated lower bound on MAE. Indeed, extensive parameter tuning efforts were unable to improve the performance of the model.

### 4.2 Automobile MPG data

**Fig 7:**
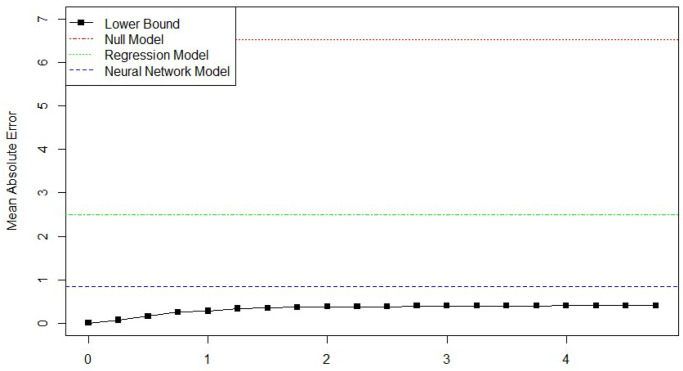
Minimum MAE as a function of *δ* for the automobile MPG study.

Next, bounds were generated for the prediction performance of machine learning models on the automobile-MPG dataset from the UCI machine learning library [34]. This is a commonly used dataset for machine learning papers, with typically good performance reported on out-of sample predictions, with MAEs as low as 2.02 MPG [35]. It contains information on the weight, acceleration, horsepower, model year, continent of origin, and fuel economy of 392 cars. The null model’s error, using the sample median as the prediction for each car, is 6.524 MPG, and a simple linear regression gives an MAE of 2.5 MPG. The best machine learning model found was a neural network with an MAE of 0.90. Generating the lower bound as a function of *δ* (summarized in Fig 5), found a lower bound of 0.85 on the MAE, using the L2 norm as the distance function, and the 5.70 × *D*(*i*, *j*) as the linking function derived from a simple linear regression model on the normalized data (each standard deviation of weight decrease fuel economy by 5.7 MPG). In this case, *δ* = 1.25 was chosen as the similarity threshold, as this is the smallest value that satisfies the 5% rule of thumb. This implies that there is little room to improve the neural network model with a reported MAE of 0.90, compared to the estimated lower bound of 0.85. Given these car characteristics, 87% of the car’s MPG is predictable, while the remaining 13% is explained either by other factors, or by idiosyncrasies of each car. The results of this study are shown in Fig 7.

### 4.3 Stock returns

Last, the use of the performance bound methodology on a large dataset of historical stock prices is demonstrated. A bound is generated using ten thousand randomly selected stock quarters from the NYSE between 2000 and 2010. The outcome of interest is the fractional change in value of the stock based on financial fundamentals. Using normalized input vectors of the stocks’ income, liabilities, earnings per share, and stock price, the model predicts how much a given amount of money invested in a stock at the start of a quarter will be worth at the close of the quarter. Because the returns are skewed, the logged value is used as the outcome variable.

The linking function is *f*(*D*(*i*, *j*)) = 0.013 × *D*(*i*, *j*), because when running a linear regression on a normalized dataset, the maximum absolute coefficient is 0.013. This means that a marginal change, *ϵ*, in any input variable corresponds linearly, on average, to no more than a 0.013 × *ϵ* change in the outcome (i.e. a 1 standard deviation change in the inputs can correlate with no more than a 1.3% increase or decrease in value).

Using the L2 distance measure and a *δ* of .04, which is the smallest value that satisfies the rule of thumb derived above, that the lower bound on MAE corresponds to 24% of the total variation in the sample. The lower bound for the mean absolute error is 0.2128 log-dollars, when the initial investment in each stock is $10,000. The null model, predicting the median for each observation, has an MAE of 0.2815. This leaves the remaining 76% of the deviation in stock returns as random or only predictable using data outside these fundamentals. The fact that so little of the outcome is predictable means that there is no model that can consistently give accurate predictions. Compared to the MPG problem, where the mileage is mostly predictable, the bound methodology shows that predicting stock returns from fundamentals is a “hard” problem, with little usable information in the dataset. Indeed,the bound shows this to be true: linear regression and neural network models have mean absolute errors of 0.282 and 0.280 respectively, hardly any different than random guessing.

## 5 Discussion and limitations

This paper presents a novel method of generating a bound on the possible performance of any prediction model, given a set of input features and outcome data. It shows that the bounding methodology gives accurate bounds and behaves consistently on simulated data, and that it generates reliable estimates for maximal prediction accuracy on real world datasets. This work is relevant to many different theoretical literatures, particularly the discussion of Information Quality [5] and the bias-variance tradeoff [33]. This approach is the first to decompose how much of the error from a given machine learning model is “reducible” and how much is inherent to the problem and dataset. This paper is the first to show how to generate bounds on irreducible error for an aribtrary dataset for any machine learning methodology in the continuous prediction space. Previous research has either generated bounds for specific modelling approaches (e.g., only for nearest-neighbor classification), or has focused only on clustering and classification. This paper uses a data-driven heuristic to generate tight bounds on irreducible error that is agnostic to data type and modelling approach.

When using this method to generate an upper bound, researchers must make three choices: the distance function, the linking function, and the size of *δ* to use. In order to make this method consistent and applicable across domains and datasets, the following standard choices are proposed and are used in each of the real-world applications: using the standardized L2 norm for the distance function, using the largest absolute regression coefficient times the distance as the linking function, and using the smallest *δ* that satisfies the “5% of possible constraints or at least 5 constraints per observation” rule.

While the bounds show good performance on real-world data, there are a number of limitations. First, the method can struggle with nonlinear relationships in data, as the underlying linking function described assumes a linear relationship. Second, the method can struggle when there are large discontinuities in the dataset. Lastly, the method requires a distance function be specified, which can be hard when inputs are categorical.

This methodology is aimed at quantifying the predictability on real world datasets. However, it is possible to construct examples where the bounding methodology fails. For example, if a dataset has observations where *Y* = *X*^2^, with *X* Uniformly distributed on [−Θ, Θ], a linear regression will yield a zero regression coefficient, which would make the standard linking function *f*(*D*(*i*, *j*) = 0 × *D*(*i*, *j*), which obviously is not a reasonable linking function. This could be remedied by doing more rigorous feature generation, and including a squared term as a predictor as well. However, without having the domain knowledge or data understanding to do that, this method would fail. As an extension, the linking function could use the maximum slope of a spline model, or look just at subregions of variables for generating the linking function. However, any change that makes the linking function more generous will generate looser bounds, so there is a tradeoff between flexibility and performance.

Similarly, the method might fail if there are steep discontinuities in the outcome. If, for example a dataset has a function of the form:

$$\left\{ \begin{array}{cc} Y=-100+100\times X & \text{for}\;X\leq 0\\ Y=100+100\times X & \text{for}\;X>0 \end{array}\right.$$

then near 0, the bounding methodology will generate many incorrect constraints. This could be remedied, however, by adding in a new variable which captures the discontinuity. The constraints in the LP rely on a local smoothness assumption, that similar observations should have similar predictions. If these constraints are violated in a systemic way, as in the example above, the bound may not be accurate. If the data have consistent, sharp, discontinuities, then discrete prediction methods, like regression trees, may be able to outperform the bounds generated by this method. In these cases, a regression tree would not generate internally consistent predictions, but this would be because the underlying data were also not internally consistent.

The results presented here use MAE as the loss function to be minimized in the LP formulation. If, instead, mean squared error were the error metric of interest, the bound could be generated exactly the same, except replacing the absolute deviation function with the squared error in the objective function. This results in a convex optimization problem with linear constraints, which is similarly easy to solve. In general, any loss function can be substituted in to the objective function, and the minimization run to generate a lower bound on that loss function. The underlying constraints and assumptions on valid predictions remain the same.

Lastly, the methodology produces looser bounds when noninformative or essentially random variables are added to a dataset. If the dimensionality of the dataset increases, but the new variables contain no actual information, observations that are similar in terms of pertinent variables may have a large distance between them, if their values for the variables with no true signal are very different. This makes the constraints looser and less reflective of true trends in the data. If, for example, the color of the paint were included in the automobile MPG example, it simply would make similar cars look farther apart, and thus make the constraints looser. Adding in extraneous variables doesn’t make the bound wrong (i.e. negative looseness), like large discontinuities can, but it makes it looser and a less accurate measure of the information in the dataset, because otherwise similar data points appear farther apart due to the extraneous variables.

In most real world outputs, with naturally occurring data patterns, the bounding methodology is expected to work well. S1 Appendix shows that this rule performs well on datasets from the UCI machine learning laboratory. The simple linking function of using the absolute value of the largest regression coefficient from a standardized regression model works well in practice, but other linking functions could be used as well.

Future work could address these limitations by expanding the objective to minimize squared error, or any other function of the predictions. Additional work could be used to use the method as a feature generation or variable selection method. If removing a variable from a dataset does not significantly increase the amount of error in the bound, then that variable is not likely to have significant predictive power. Lastly, this method could be used for outlier detection or data cleaning. Points for which it is impossible to generate a close prediction, due to the constraints in the model, are points that are less predictable by the data. This my be due to a data etnry error, a missing variable, or just statistical noise, but it highlights the interesting points worth further exploration.

## 6 Conclusion

The methodology presented in this paper offers a pragmatic approach for researchers to evaluate the robustness of prediction models. A recurrent query in the realm of statistical modeling, especially from novices, often revolves around understanding the quality metrics—“what is a good value for r-squared?” or “what is a good mean forecast error?” These questions, inherently, don’t have a definitive answer. However, the method introduced—aimed at estimating the potential predictive performance—provides a dataset-specific benchmark. This permits us to offer answers tailored to both the dataset under consideration and the research question in play.

The disparity between the MAE (Mean Absolute Error) of a prediction model and the inferred MAE bound provides an insightful gauge for researchers. If the realized MAE of a model gravitates closely to this bound, then further refinement or tuning of the model might not significantly enhance its predictive capabilities. In such scenarios, endeavors might be more fruitfully channeled towards amassing more data or diverting focus to alternative research challenges. Conversely, a pronounced gap between the model’s predictive accuracy and the bound suggests the potential benefits of refining the algorithms.

Beyond model evaluation, the methodology offers a tool for gauging the intrinsic quality of a dataset. For a given outcome of interest, the juxtaposition of the MAE bound and the null model offers a yardstick to evaluate what proportion of the outcome’s variations can be foreseen leveraging the dataset. A high predictive percentage is indicative either of a high-quality dataset or a relatively simpler prediction challenge. On the flip side, a lower predictable percentage flags either a complex prediction problem or potentially a dataset that may lack in quality or pertinent information.

## Supporting information

S1 Appendix (PDF)
