# Supplementary material for: As good as it gets? A new approach to estimating possible prediction performance
Source: PLoS One. 2024 Oct 16;19(10):e0296904. doi: 10.1371/journal.pone.0296904 (PMC11482679; doi:10.1371/journal.pone.0296904)
Supplement: S1 Appendix — (PDF) [file pone.0296904.s001.pdf]

Appendix A: Test Datasets

The bounds performance and behavior were analyzed for five datasets: the three from the body of the paper, and two wine quality datasets from the UCI machine learning depository. In each of these examples, the standardized methodology is used: using the L2 norm on standardized input variables, and using $\beta_{\max} \times D(i, j)$ as the linking function. Table 2 summarizes the MAE bound, the MAE of the null model, the MAE of a neural network model, and the smallest value of δ that meets the 5% rule of thumb.

[Table 2 about here.]

The UCI repository contains scores on wine quality for red and white Portuguese wines, and information on the physiochemical aspects of the wine (e.g. acidity, density, residual sugar, alcohol level, etc.). The prediction task is to estimate the wine quality score as a function of its chemical composition. Because of the differences in the wines, the two wine types (red and white) are split into separate datasets. The largest absolute regression coefficient is -0.44949, so the linking function is $f(D(i, j)) = 0.4494 \times D(i, j)$. Figs A1-A9 shows the minimum achievable mean absolute errors, using the L2 norm for the distance function for a range of values of δ.

The smallest value for δ that satisfies the 5% rule is 2.4, which corresponds to a minimum achievable MAE of 0.334. The null model has a MAE of 0.635, the best machine learning model found, a regression tree, had a MAE of 0.464.

The results for red wine quality are similar. The largest absolute regression coefficient is 0.29433, and the $L2$ norm is used for the distance function. The resulting bound curve as a function of δ is shown in Figs A1-A9. The smallest δ value that satisfies the 5% rule is 2.1. This gives a minimum achievable MAE of 0.354, compared to 0.6579 for the null model, and 0.410 for the best machine learning model.

List of Figures

A1 The performance bound (a) and the Looseness (b) as a function of δ and σ when N=750 and M=4. 18
A2 Looseness as a function of (a) δ and M (N=750, and $\sigma = 3$) and (b) N and M ($\sigma = 3$, and $\delta = 3$) 19
A3 The looseness of the bound compared to the true model, as N and σ change, using the 5% rule of thumb to choose δ 20
A4 Looseness of the bound as a function of the size of discontinuities in the data and noise 21
A5 Looseness of the bound as a function of the size of interaction and squared terms in the data generating process 22
A6 Minimum MAE as a function of δ for the technology adoption study. . . 23
A7 Minimum MAE as a function of δ for the automobile MPG study. . . . 24
A8 White wine MAE lower bounds compared to the null model and a regression tree 25
A9 Red wine MAE lower bounds compared to the null model and the best machine learning model 26

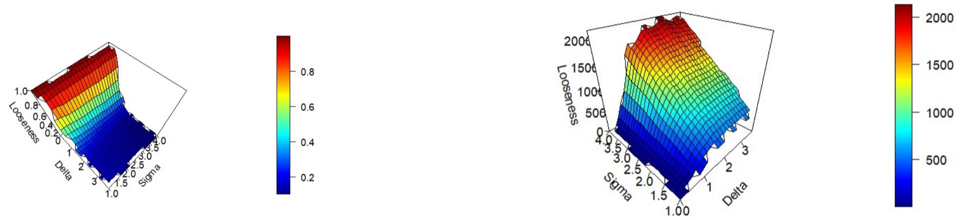

Fig A1. The performance bound (a) and the Looseness (b) as a function of δ and σ when N=750 and M=4.

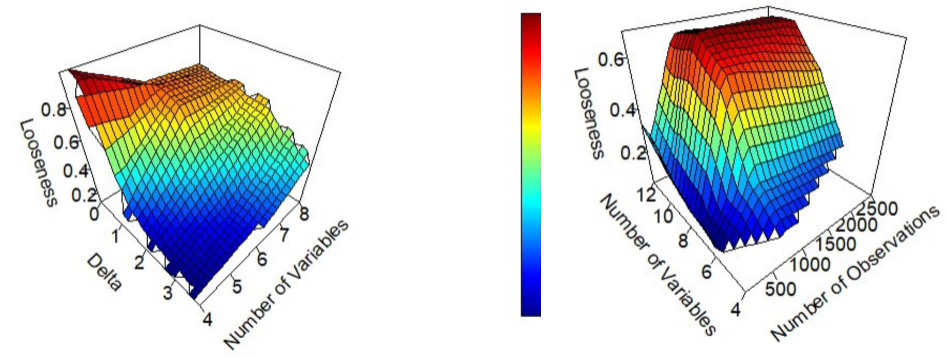

Fig A2. Looseness as a function of (a) δ and M (N=750, and $\sigma = 3$) and (b) N and M ($\sigma = 3$, and $\delta = 3$)

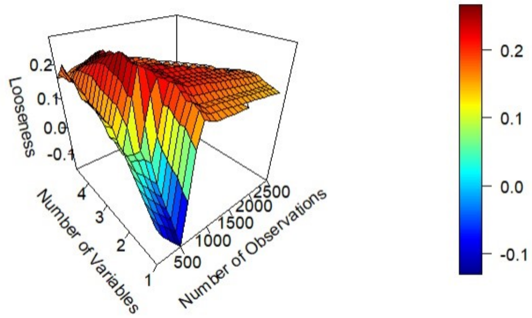

Fig A3. The looseness of the bound compared to the true model, as N and σ change, using the 5% rule of thumb to choose δ

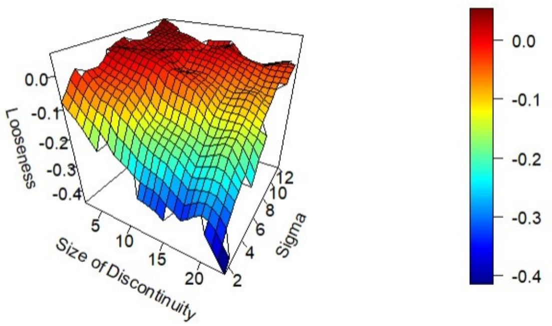

Fig A4. Looseness of the bound as a function of the size of discontinuities in the data and noise

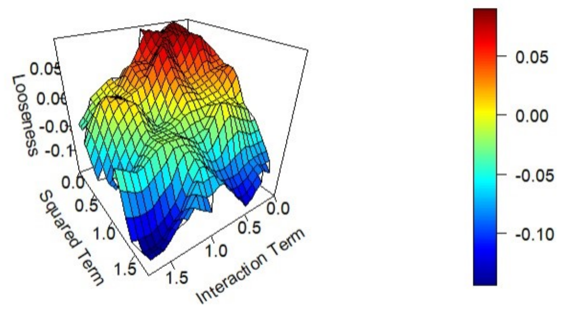

Fig A5. Looseness of the bound as a function of the size of interaction and squared terms in the data generating process

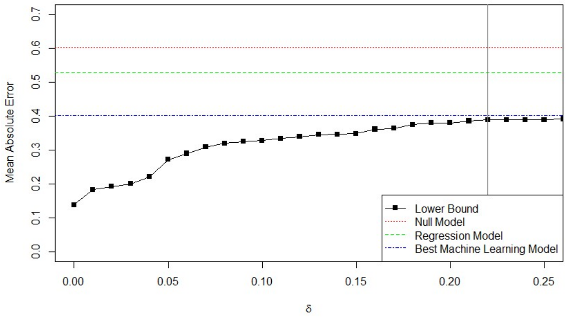

Fig A6. Minimum MAE as a function of δ for the technology adoption study.

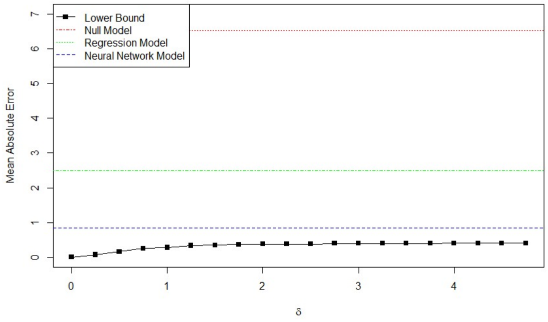

Fig A7. Minimum MAE as a function of δ for the automobile MPG study.

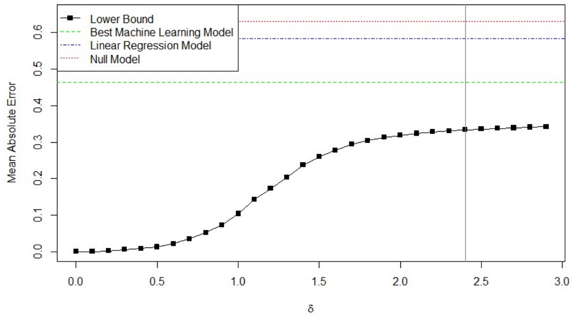

Fig A8. White wine MAE lower bounds compared to the null model and a regression tree

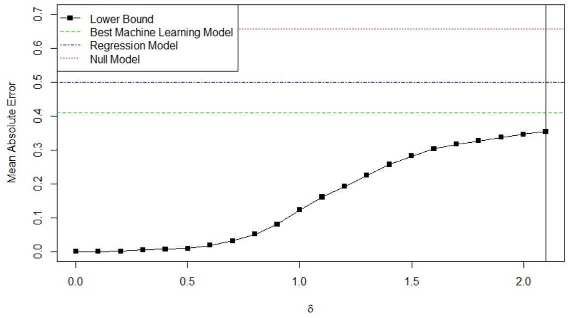

Fig A9. Red wine MAE lower bounds compared to the null model and the best machine learning model

List of Table

2 Summary of different datasets 29

Table 2. Summary of different datasets

Dataset	MAE_Bound	δ-rule	MAE_Null	MAE_Model	ϕ	Predictive Score
Red Wine	0.354	2.1	0.6579	0.410	46.2%	81.6%
White Wine	0.340	2.4	.6305	0.464	47.0%	57.3%
Stocks	0.217	0.09	0.2815	0.280	22.9%	2.3%
MPG	0.847	1.25	6.524	0.900	87.2%	99.1%
Tech Adoption	0.369	0.70	0.602	0.46	38.7%	60.9%
